# Fetal Cervical Neuroblastoma: Prenatal Diagnosis

**DOI:** 10.1155/2011/529749

**Published:** 2011-08-04

**Authors:** Ismail Güzelmansur, Hatice Tatar Aksoy, Sibel Hakverdi, Mustafa Seven, Uğur Dilmen, Gülçin Dilmen

**Affiliations:** ^1^Department of Radiology, Hatay Mozaik Hospital, 06100 Hatay, Turkey; ^2^Department of Neonatology, Zekai Tahir Burak Women's Health Education and Research Hospital, Sıhhiye, 06100 Ankara, Turkey; ^3^Department of Pathology, Faculty of Medicine, Mustafa Kemal University, 06100 Hatay, Turkey; ^4^Department of Obstetric and Gynecology, Hatay Mozaik Hospital, 06100 Hatay, Turkey; ^5^Department of Radiology, Fatih University School of Medicine, 06100 Ankara, Turkey

## Abstract

Neuroblastoma is the most frequent extracranial solid tumor in childhood, but it is seldom diagnosed prenatally. It usually presented with adrenal masses. Presentation of other localization is extremely rare. We report a case of cervical neuroblastoma identified at 20 weeks of gestational age. This is the third case diagnosed antenatally on neck region in the literature. Additionally, it is the first case that extended to the brain. We also discussed the literature for cervical neuroblastoma detected prenatally.

## 1. Introduction

Fetal cervical neuroblastoma is an extremely rare condition that should be considered in the presentation of fetal solid neck masses [1, 2]. Differential diagnosis of sonographically detected fetal neck tumours is difficult. Encephalomyelocele, lymphangioma/hygroma, teratoma, sarcoma, haemangioma, neuroblastoma, and goitre should be in mind for differential diagnosis of the fetal neck masses [3]. We present a fetal cervical neuroblastoma detected by prenatal sonography at 20 weeks gestation. We would like to draw attention to this case since this is the third case diagnosed antenatally in the neck region and the first case extended to the brain. We also discussed the cases of fetal cervical neuroblastoma detected by prenatal sonography in the literature.

## 2. Case Report

**Figure 1:**
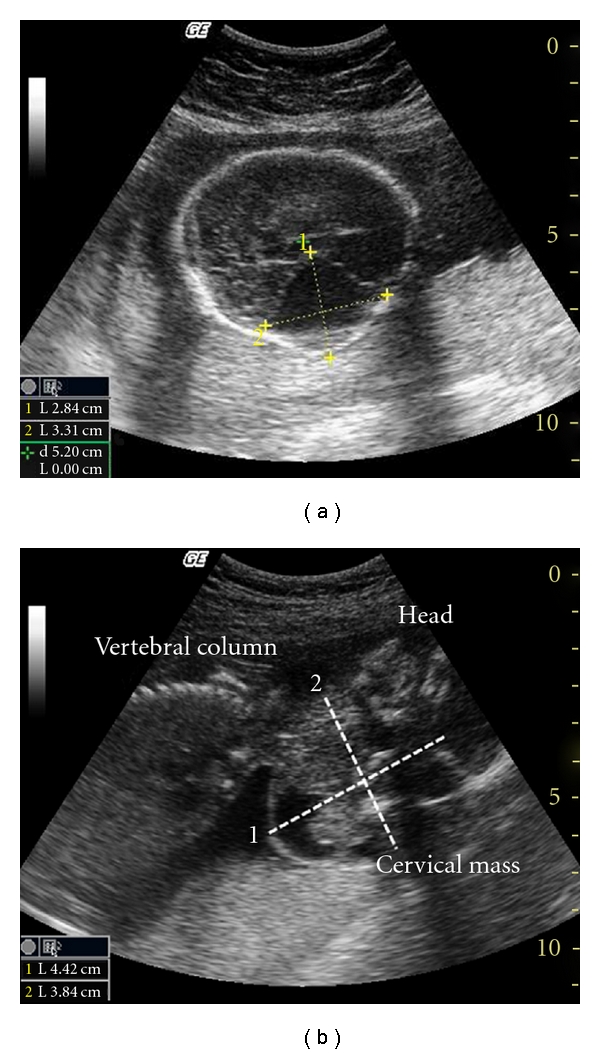
(a) The mass extending to the brain. (b) The mass in the cervical region.

A 32-year-old woman (gravida 2, para 1) was referred for evaluation of a fetal neck mass, which had been identified on routine sonography at 20 weeks' gestation. Family history revealed that the father was 35 years old and no consanguinity. The detailed ultrasonograhic evaluation revealed a fetus with a 20 weeks appearance and weighed 372 gr. Left lateral neck mass was observed with sized 49 × 38 mm that starts from preauricular region to left mandibular site. The mass was hyperechoic, mainly solid, and contained few cystic components. There were destructive lesions on left mandibula, maxilla, and sphenoid bone. It was extended to the brain. (Figures 1(a) and 1(b)).

An abortion was performed at 20 weeks and 5 days of gestation with parents consent.

**Figure 2:**
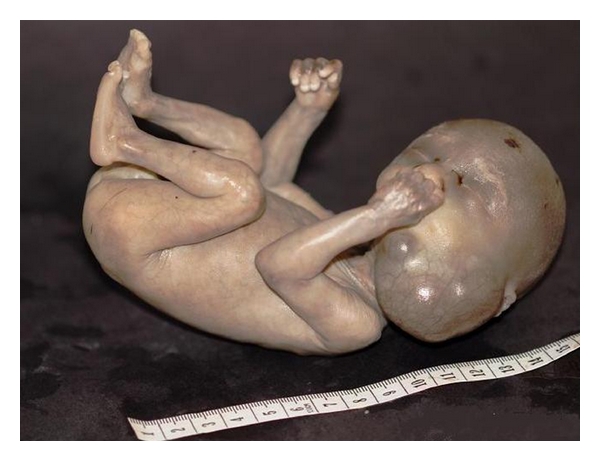
The macroscopic appearance of the aborted fetus.

The macroscopic appearance of fetus is showed on Figure 2.

**Figure 3:**
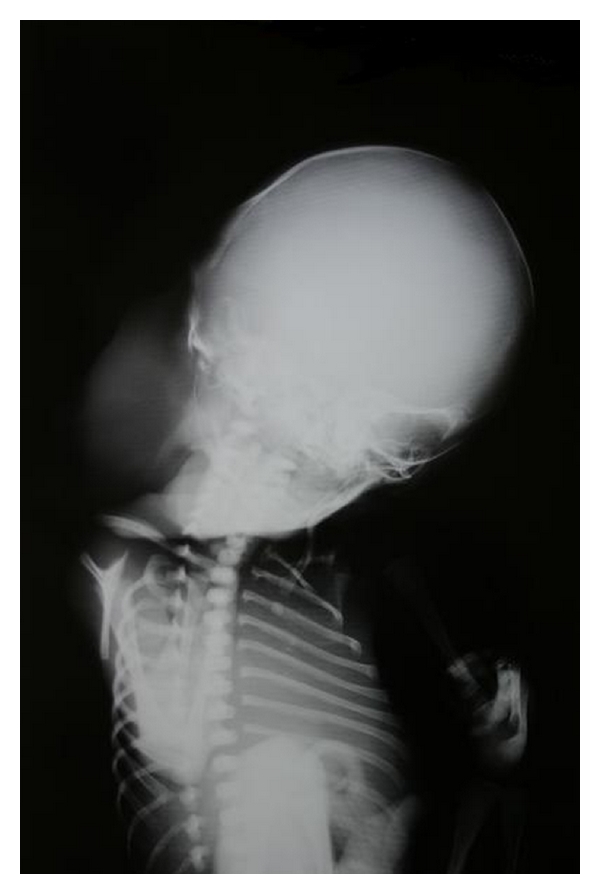
X-ray of the aborted fetus.

Aborted fetus was examined with X-ray. It showed cervical mass in Figure 3.

**Figure 4:**
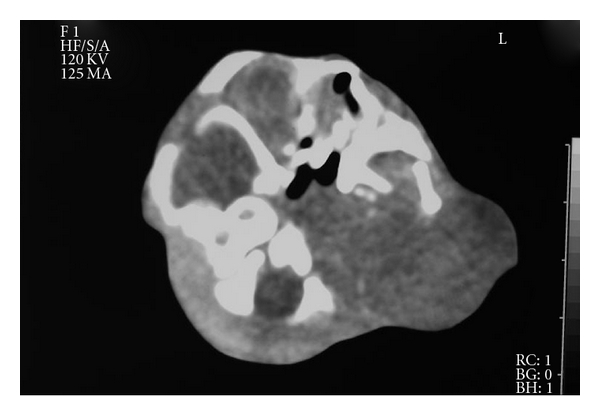
CT image of the cervical mass of aborted fetus.

Computerized tomography of the head and neck region revealed no extension of the mass into thorax but extension into the brain shown in Figure 4.

**Figure 5:**
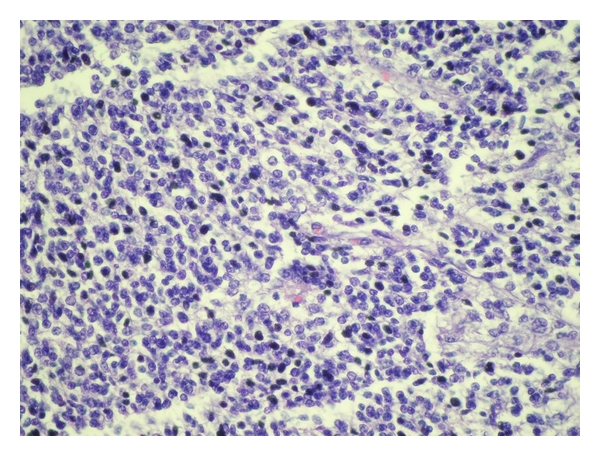
Histopathologic examination of the mass.

Histopathological examination of the tumor was consistent with neuroblastoma which was shown in Figure 5. Immunohistochemical staining for keratin, smooth muscle actin, vimentin, and desmin was negative. Shimada classification and N-myc amplification were not assessed.

The mother had none of the following conditions during the course of her pregnancy: maternal exposure to hydantoin, phenobarbital, alcohol, teratogenic agents or radiation, familial neuroblastoma, and maternal diabetes mellitus. There was no family history of malformations. Blood pressure of mother was within normal limits during pregnancy; she did not experience any infection or disease during pregnancy. She did not take any medication and her tests for fetal anomalies were also within normal limits.

## 3. Discussion

The incidence of neonatal neuroblastoma has been reported to be 0.61 per 100.000 births [2]. The majority of prenatally detected neuroblastomas are apparently isolated and located in the adrenal.

Prenatal diagnosis of neuroblastoma by ultrasound was first reported in 1983 [4]. Ultrasound images identify a retroperitoneal mass, separate from the liver, and superior to the kidney as >90% occur in the adrenal gland. It is cystic, and around 50% have solid components; solid masses are more likely to metastasize to the liver. Adrenal hemorrhage often complicates neuroblastoma and can delay diagnosis. Only half of suprarenal masses are neuroblastomas, so other lesions such as extralobar sequestration should be considered. The presence of calcification is also sonographically useful, and its detection has been associated with improved survival, presumably indicating previous tumor necrosis.

Fetal cervical neuroblastoma is an extremely rare condition that should be considered in the presentation of fetal solid neck masses. In a recent review, a total of 271 fetuses and neonates presented with neuroblastoma and 41.3% of them were diagnosed prenatally (*n* = 112). Only two neuroblastomas were diagnosed prenatally on neck region in this report. The most common initial finding was a mass detected either by ante- or postnatal sonography or by physical examination during the neonatal period with signs and symptoms referable to the location of origin [1]. The extention to the brain is very rare. Our case was the first case that extended into the brain.

Gorincour et al. reported a case of cervical neuroblastoma. It was a 2100 g baby boy delivered by cesarean section at 33 weeks of gestation after premature labor which resulted in fetal distress. Death occurred on day 3 as a result of multiorgan failure and disseminated intravascular coagulation. Prenatally it was suggested as teratoma but after delivery and initial clinical stabilization biopsy of the cervical mass, neuroblastoma was revealed [5].

Differential diagnosis of sonographically detected fetal neck tumours is difficult. Lymphangiomas, teratomas, and hemangiomas can occur in the neck region. In the anterior neck, other masses can be caused by soft-tissue lesions, such as sarcomas, hamartomas (as can be seen in rare cases of tuberous sclerosis), or fetal goiter. Masses in the anterior neck may cause hyperextension of the neck when large and are frequently associated with polyhydramnios [3]. Our case was a fetal solid neck mass suggestive of teratoma, which proved postnatally to be a neuroblastoma. Beside the ultrasonography and magnetic resonance image, elevated catecholamine metabolites are helpful for diagnosis of neuroblastoma prenatally. Elevated catecholamine metabolites are present in >90% of cases and can cause antenatal symptoms such as maternal hypertension. Twenty-four-hour maternal urine assay or amniocentesis for fetal urinary catecholamine metabolites (VMA-HVA) can be helpful when the prenatal diagnosis of neuroblastoma is unclear or the cause of maternal hypertension is suspected [6]. In our case, the mother did not have hypertension. There is currently no indication for fetal intervention in fetal neuroblastoma. Serial ultrasound can be used to evaluate tumor size and the possibility of metastatic liver enlargement. Additional investigations, including MRI, can be helpful. Cervical tumours may cause severe airway compression, and an airway cannot be established promptly. Workup of solid fetal cervical masses should include fetal MRI to evaluate the airway compromise. We did not evaluate the airway compression by MRI.

Cesarean section can be utilized if there appears to be an increased risk for dystocia or tumor rupture.

Cervical neuroblastomas should be listed in the differential diagnosis of fetal neck mass that was determined by ultrasonography.

## References

[1] Isaacs H (2007). Fetal and neonatal neuroblastoma: retrospective review of 271 cases. *Fetal and Pediatric Pathology*.

[2] Acharya S, Jayabose S, Kogan SJ (1997). Prenatally diagnosed neuroblastoma. *Cancer*.

[3] Kazan J, Levine D (2007). Imaging of fetal tumors. *Ultrasound Clinics of North America*.

[4] Fénart D, Deville A, Donzeau M, Bruneton JN (1983). Retroperitoneal neuroblastoma diagnosed in utero: a propos of 1 case. *Journal de Radiologie*.

[5] Gorincour G, Dugougeat-Pilleul F, Bouvier R (2003). Prenatal presentation of cervical congenital neuroblastoma. *Prenatal Diagnosis*.

[6] Sebire NJ, Jauniaux E (2009). Fetal and placental malignancies: prenatal diagnosis and management. *Ultrasound in Obstetrics and Gynecology*.

